# Risk factors and prediction model development for pancreatic fistula following splenectomy in Wilson’s disease patients with portal hypertension

**DOI:** 10.1186/s12876-025-04475-w

**Published:** 2025-11-25

**Authors:** Zhou Zheng, Yi Shen, Hui-cong Min, Hui Peng, Long Huang, Wan-zong Zhang, Hui Feng, Qing-sheng Yu

**Affiliations:** 1 Anhui University of Chinese Medicine, 350 Longzi Lake Road, Anhui, China; 2 The First Affiliated Hospital of Anhui University of Chinese Medicine, 117 Mei Shan Road, Hefei, Anhui 230031 China; 3 The Second Affiliated Hospital of Anhui, University of Chinese Medicine, 300 Shou Chun Road, Hefei, Anhui China; 4 The Third Affiliated Hospital of Anhui University of Chinese Medicine, 45 Shi He Road, Hefei, Anhui China

## Abstract

**Objective:**

Postoperative pancreatic fistula (POPF) following splenectomy in patients with Wilson’s disease (WD) complicated by portal hypertension adversely affects prognosis. Therefore, developing and validating an individualized nomogram to predict POPF risk is crucial.

**Methods:**

This retrospective study included 519 patients with WD-associated splenomegaly and hypersplenism who underwent splenectomy between January 2016 and June 2025. Patients were divided by center into a training cohort (*n* = 411) and a validation cohort (*n* = 108). Least absolute shrinkage and selection operator (LASSO) regression was used to select candidate variables, which were then incorporated into multivariable logistic regression to construct a predictive model and develop a nomogram. Shapley additive explanations (SHAP) were applied to interpret the selected variables. Restricted cubic spline (RCS) curves were used for threshold analysis of continuous variables. Model discrimination was evaluated using the area under the receiver operating characteristic curve (AUC). Calibration was assessed with calibration curves and the Hosmer-Lemeshow (HL) test, while clinical net benefit was determined via decision curve analysis (DCA). Predictive performance was compared with preoperative composite indices, including C-reactive protein to albumin ratio (CAR), neutrophil-to-lymphocyte ratio (NLR), and platelet-to-lymphocyte ratio (PLR).

**Results:**

The overall incidence of POPF was 18.3% (95/519). LASSO regression identified seven variables: operation time, history of abdominal surgery, type of splenic hilum division, body mass index (BMI), splenomegaly grade, main pancreatic duct (MPD) diameter, and Clavien-Dindo classification (CDC) complications. Multivariable analysis revealed operation time (OR = 1.039), history of abdominal surgery (OR = 2.223), BMI (OR = 1.078), grade III splenomegaly (OR = 2.521), and CDC complications (OR = 1.726) as risk factors; type II splenic hilum division (OR = 0.494) and MPD diameter > 3 mm (OR = 0.509) as protective factors (all *P* < 0.05). SHAP interpretation indicated that operation time (+ 0.075) and history of abdominal surgery (+ 0.059) were the primary contributors to POPF prediction. RCS analysis highlighted BMI exceeding 24 kg/m² and operation time exceeding 200 min as key thresholds for increased POPF risk. The nomogram demonstrated AUC values of 0.761 (95% CI: 0.697–0.825) in the training cohort and 0.814 (95% CI: 0.702–0.927) in the validation cohort. The HL test yielded *P* = 0.615 and *P* = 0.410, respectively, indicating good calibration. DCA showed high net benefit in the training cohort at threshold probabilities of approximately 12%-91% and in the validation cohort at 15%-98%. Compared with CAR, NLR, and PLR, the nomogram exhibited superior AUC (0.761 vs. 0.621, 0.662, 0.633; DeLong test, all *P* < 0.05).

**Conclusion:**

The nomogram, constructed from a multivariable model, effectively discriminates and calibrates POPF risk following splenectomy in patients with WD, offering potential clinical utility. Particular attention should be paid to history of abdominal surgery and operation time, with preoperative individualized interventions tailored accordingly.

**Supplementary Information:**

The online version contains supplementary material available at 10.1186/s12876-025-04475-w.

## Introduction

Wilson’s disease (WD), a rare autosomal recessive disorder caused by ATP7B gene mutations, disrupts copper metabolism and results in pathological copper accumulation in the liver, brain, and corneas. This accumulation induces mitochondrial, nucleic acid, protein, and lipid damage within hepatocytes, ultimately presenting as chronic hepatitis, cirrhotic portal hypertension with splenomegaly and hypersplenism, and neuropsychiatric manifestations [1]. The estimated global prevalence ranges from 1/10,000 to 1/30,000, with European cohorts demonstrating higher rates (12–29/100,000) [2, 3]. First-line therapies involve copper chelators (e.g., D-penicillamine, 2, 3-dimercaptopropane-1-sulfonate) and zinc supplements to enhance copper excretion. However, these agents frequently induce myelosuppression, exacerbating leukopenia and thrombocytopenia [4]. Concurrently, cirrhosis-induced portal hypertension worsens splenomegaly and hypersplenism, further deteriorating hematologic parameters and compromising the safety and sustainability of anticopper therapy [5].

Since the 1990 s, splenectomy combined with intensified anticopper regimens has been reported to ameliorate cytopenia and hypersplenism, thereby restoring leukocyte/platelet counts and enabling sustained copper-depletion therapy [6]. Nevertheless, splenectomy carries intrinsic risks: anatomical proximity between the splenic and pancreatic tails predisposes to iatrogenic pancreatic injury during hilar dissection, potentially triggering postoperative pancreatic fistula (POPF) [7]. Aligning with International Study Group on Pancreatic Surgery (ISGPS) criteria, contemporary studies report an overall POPF incidence of 14.6% post-splenectomy-comprising biochemical leaks (BL, 10.1%) and clinically relevant POPF (Grade B/C, 4.5%) [8]. POPF prolongs hospitalization, escalates therapeutic interventions, delays recovery, and adversely impacts long-term prognosis [9].

Existing POPF research predominantly focuses on pancreatectomy. Systematic reviews and multicenter analyses consistently identify soft pancreatic texture, elevated BMI, intraoperative blood loss, transfusion requirements, and prolonged operation time as key independent risk factors [10]. Some studies also correlate POPF risk with postoperative drain amylase levels and inflammatory indices (e.g., CRP/albumin ratio) [11]. However, no studies address POPF specifically after splenectomy in WD patients with portal hypertension—a population uniquely vulnerable to both surgical and metabolic complications. This study aimed to identify key risk factors for POPF in patients with Wilson’s disease complicated by portal hypertension undergoing splenectomy, and to construct a concise and practical clinical prediction model. Concurrently, threshold analysis of pivotal indicators was performed to enhance the model’s predictive accuracy, thereby providing decision-making support for preoperative screening, precise interventions, and improved prognosis.

## Materials and methods

### Study population

A total of 519 patients with WD, splenomegaly, and hypersplenism who underwent splenectomy at the Affiliated Hospitals of Anhui University of Chinese Medicine between January 2016 and June 2025 were retrospectively enrolled. Patients were stratified by medical center. The training cohort comprised 411 patients from the First Affiliated Hospital, with a median age of 27 years (215 males, 196 females). POPF occurred in 74 cases (Grade A: 50; Grade B: 15; Grade C: 9), while 337 patients did not develop POPF. The validation cohort consisted of 108 patients from the Third Affiliated Hospital. Post-splenectomy, pancreatic fistula was observed in 95 patients in two medical centers, including 47 (9.06%) cases of biochemical leak (BL), 39 (7.51%) cases of grade B fistula, and 9 (1.73%) cases of grade C fistula. Notably, 9 grade B fistulas progressed from BL, and 1 grade B fistula progressed to grade C. Among the grade C fistulas, 6 patients recovered and were discharged following treatment; however, 3 patients died despite therapeutic interventions (Suppl. 1).

WD was diagnosed according to the following criteria: (1) markedly reduced serum ceruloplasmin, typically < 20 mg/dL; (2) significantly elevated 24-hour urinary copper excretion, usually >100 µg/day; (3) presence of Kayser–Fleischer rings at the corneal margin on slit-lamp examination; (4) hepatic copper content >250 µg/g dry weight; and (5) identification of pathogenic ATP7B gene mutations [12].

Splenomegaly grading was defined as follows: Grade I, spleen palpable 2–3 cm below the left costal margin; Grade II, spleen extending >3 cm below the costal margin but not reaching the umbilicus; Grade III, spleen extending beyond the umbilical level and possibly reaching the midline [13].

Hypersplenism grading was defined as: Mild, leukocytes 3.0–4.0 × 10⁹/L, erythrocytes 2.5–3.5 × 10¹²/L, platelets 7.0–10.0 × 10⁹/L; Moderate, leukocytes 2.0–3.0 × 10⁹/L, erythrocytes 1.5–2.5 × 10¹²/L, platelets 5.0–7.0 × 10⁹/L; Severe, leukocytes < 2.0 × 10⁹/L, erythrocytes < 1.5 × 10¹²/L, platelets < 5.0 × 10⁹/L [14].

POPF was defined according to the 2016 International Study Group on Pancreatic Surgery (ISGPS) criteria as drain fluid amylase ≥ 3 times the upper limit of normal serum amylase on or after postoperative day 3, or/and requiring clinical management. Grade A (Biochemical Leak): Drainage fluid amylase concentration ≥ 3 times the upper normal limit, with no clinical symptoms and no need for intervention, resulting in only a slight increase in hospital stay and costs. Grade B: Drainage fluid amylase concentration ≥ 3 times the upper normal limit, accompanied by clinical symptoms or complications (e.g., fever, abdominal pain, effusion), requiring prolonged drainage placement (>3 weeks), antibiotic use, or other interventions. Grade C: Drainage fluid amylase concentration ≥ 3 times the upper normal limit, associated with severe intra-abdominal infection or necrosis, hemorrhage (postoperative pancreatic bed bleeding), multiple organ dysfunction, shock, necessitating ICU management, reoperation, or leading to death [15]. In our clinical practice following splenectomy, drainage tubes are routinely placed in all patients. Drainage fluid amylase levels are routinely assessed on postoperative day 3. An elevation exceeding three times the upper limit of normal is regarded as a positive event for postoperative pancreatic fistula (encompassing grades A, B, and C).

Postoperative complications were graded I-V according to the CDC [16]: Grade I, minor complications not requiring pharmacologic therapy; Grade II, requiring pharmacologic therapy; Grade III, requiring surgical or radiologic intervention; Grade IV, life-threatening complications requiring ICU management (IVa, single-organ failure; IVb, multi-organ failure); Grade V, death.

Inclusion criteria were: (1) definite diagnosis of WD; (2) hypersplenism confirmed by ultrasonography, hematological tests, or bone marrow examination; (3) liver function classified as Child-Pugh grade A or B; (4) voluntary consent to surgery; and (5) absence of severe comorbidities, with adequate general condition to tolerate surgery.

Exclusion criteria were: (1) intraoperatively confirmed pancreatic injury; (2) severe concomitant injury to the liver, intestine, or kidney (American Association for the Surgery of Trauma [AAST] grade ≥ III), which could independently cause intra-abdominal infection or inflammatory complications; (3) pregnancy or severe immunosuppression (e.g., HIV/AIDS, post–organ transplantation); (4) death within 72 h postoperatively; and (5) pre-existing pancreatic disease or prior pancreatic surgery.

### Surgical procedure

Both laparoscopic and open splenectomy were performed under general anesthesia. Prophylactic antibiotics were administered starting 5 days preoperatively.

Laparoscopic splenectomy: Patients were placed in the right lateral or semi-lateral decubitus position. Pneumoperitoneum was established via multiple trocar insertions. The splenocolic, gastrosplenic, and splenorenal ligaments were sequentially divided. After exposure of the splenic hilum, vascular control was achieved using either a linear stapler (Ethicon PROXIMATE^®^ TX60G, Green Reload; Ethicon) or an energy device (LigaSure Atlas LS1020; Covidien). The spleen was retrieved within an endoscopic specimen bag through an auxiliary incision.

Open splenectomy: A left subcostal incision was typically employed. Under direct vision, perisplenic ligaments were dissected and the splenic hilum was exposed. Vascular division was performed using the same linear stapler or energy device as described above, followed by complete splenectomy. Both surgical procedures involved routine postoperative placement of abdominal drainage tubes.

**Fig. 1:**
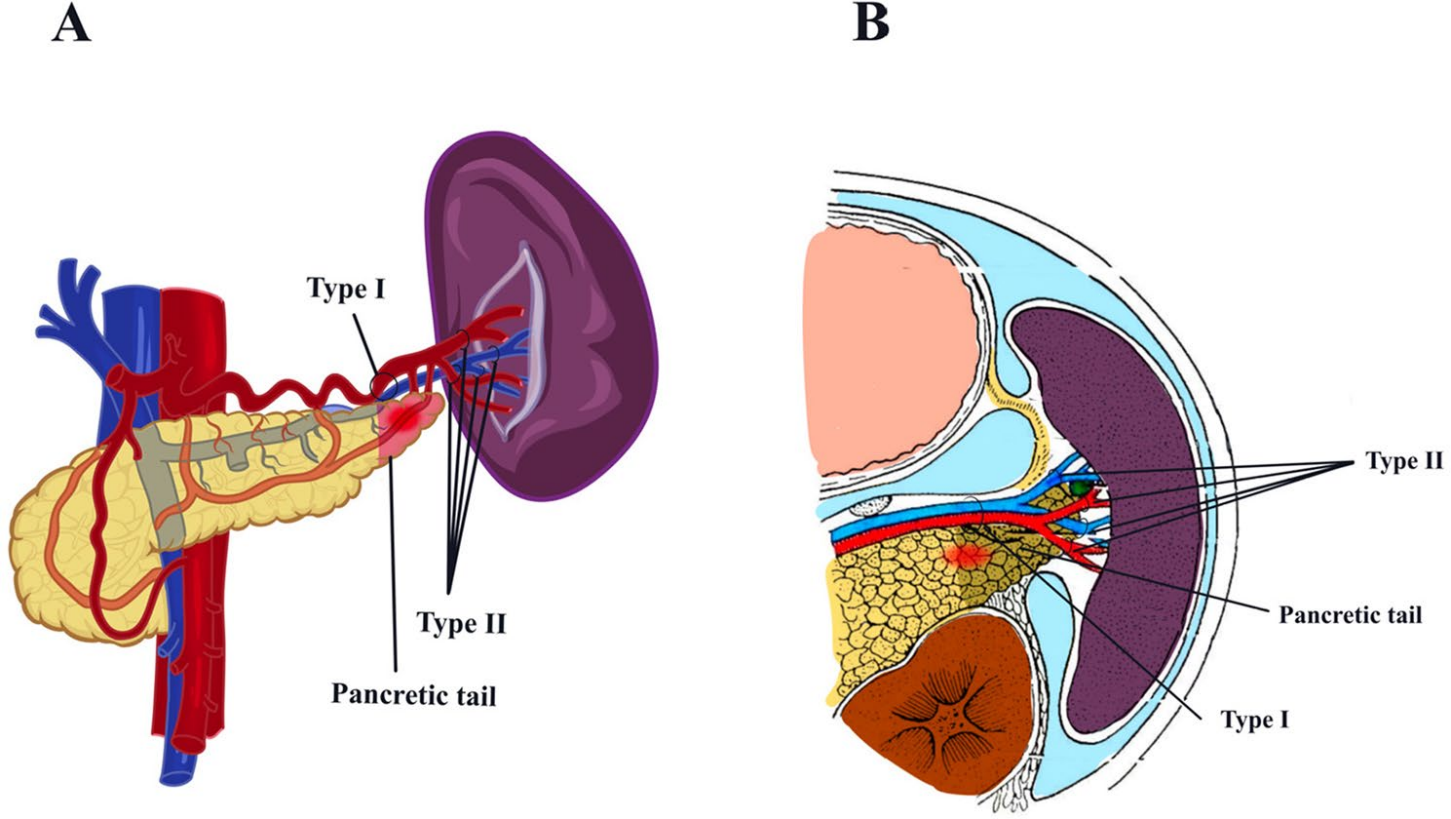
The management of vessels at the splenic hilum. Notes: Panels **A** (coronal view) and **B** (transverse section) illustrate the Type I and Type II techniques for splenic hilum division, delineating the approaches to splenic vessel management and the potential sites of pancreatic tail injury

The management of the splenic hilum is depicted in Fig. 1: Type I hilum division involves ligation of the main trunks of the splenic vein and artery in proximity to the pancreatic tail; Type II hilum division entails bundle ligation of the superior and inferior polar vessels of the splenic artery and vein at the hilum, as close to the spleen as feasible.

### Clinical characteristics and laboratory parameters

Clinical parameters were extracted from the institutional case database and included demographic and medical variables. Age and sex were recorded, while liver function was assessed using the Child-Pugh score, classified as Grade A (5–6 points), Grade B (7–9 points), or Grade C (10–15 points). Body mass index (BMI) was calculated as weight in kilograms divided by height squared in meters. Additional covariates comprised histories of diabetes mellitus, hypertension, and smoking, the latter defined as the habitual consumption of at least one cigarette per day for six consecutive months. Previous abdominal surgery and the clinical grade of splenomegaly were also documented.

Quantitative assessment of the maximum diameter of the mid-portion of the pancreatic duct was performed using multi-detector computed tomography (Siemens SOMATOM Definition Flash), with thin-slice scanning (≤ 1.25 mm) and multiplanar reconstruction. Hemodynamic parameters-including portal vein diameter (PVD), splenic vein diameter (SVD), and portal venous flow velocity (PVV)-were measured with color Doppler ultrasonography (ACUSON Antares, Siemens) employing a 5.0-MHz convex transducer. Hematologic indices were obtained on an automated hematology analyzer (Sysmex XN-9000): white blood cells (WBC), red blood cells (RBC), and platelets (PLT) were quantified by impedance-based cell counting, whereas hemoglobin (HGB) levels were determined by the sodium lauryl sulfate (SLS) method. D-dimer levels were m easured by a latex-enhanced immunoturbidimetric assay. Biochemical markers were analyzed with an automated chemistry system (Hitachi 7600-010): Alanine aminotransferase (ALT) and aspartate aminotransferase (AST) were determined by enzymatic rate methods; albumin (ALB) by the bromocresol green method; total bilirubin (TBIL) by the diazonium salt method; Ceruloplasmin (CP) was determined using immunoturbidimetric assay; blood urea nitrogen (BUN) by the urease–glutamate dehydrogenase reaction; serum creatinine (Cr) by the enzymatic creatininase method, and serum amylase activity by an enzymatic kinetic assay. Serum copper (S-Cu) and urine copper (U-Cu) were determined by atomic absorption spectrometry using a Hitachi series atomic absorption spectrophotometer.

Surgical variables were abstracted from operative records and included surgical approach (laparoscopic splenectomy [LS] or open splenectomy [OS]), splenic hilum management (energy device [ED] or non-energy dissection [NED]), hilum division technique (Type I or II), operation time, and intraoperative blood loss. Postoperative outcomes comprised POPF, diagnosed according to the 2016 ISGPS criteria (drain fluid amylase ≥ 3 times the upper limit of serum amylase on or after postoperative day 3), and postoperative complications graded by the CDC. Complication grading was independently evaluated by attending surgeons with at least five years of clinical experience.

### Calculation of composite indicators

The following composite hematological indices were calculated: CAR (C-reactive protein-to-albumin ratio): CRP concentration (mg/L)/serum albumin level (g/L); NLR (neutrophil-to-lymphocyte ratio): absolute neutrophil count (×10⁹/L)/absolute lymphocyte count (×10⁹/L); PLR (platelet-to-lymphocyte ratio): platelet count (×10⁹/L)/absolute lymphocyte count (×10⁹/L) [17, 18].

### Statistical analysis

Statistical analyses were conducted using GraphPad Prism (version 10.5.0; San Diego, USA), R Studio (version 4.4.1; Vienna, Austria) and Python (version 3.10.4, Python Software Foundation, United States). Continuous variables with a normal distribution were expressed as mean ± standard deviation (SD) and compared using independent-samples t tests. Non-normally distributed continuous data were reported as median with interquartile range (IQR; P25-P75) and analyzed using the Mann–Whitney U test. Categorical variables were presented as frequencies (%) and compared using the chi-square or Fisher’s Exact test.

LASSO regression was performed with the glmnet package, applying 10-fold cross-validation to identify potential predictors. Significant variables were subsequently entered into a multivariate logistic regression model to determine independent risk factors and construct a prognostic model. The nomogram was constructed using the “rms” package in R software, whereas the SHAP analysis was conducted via the “shap” library in Python. Restricted cubic spline (RCS) curves were plotted for the selected continuous variables with the “ggplot2” package, followed by threshold effect analysis. Model performance was evaluated in terms of discrimination, calibration, and clinical utility. Discrimination was assessed by ROC curves using the “pROC” package, with the AUC calculated to quantify predictive accuracy. Differences in AUC values between the nomogram and composite indicators were compared using DeLong’s test. Calibration was assessed with calibration plots generated by the “rms” package, and clinical utility was evaluated using DCA with the “rmda package”. A two-sided *P* value < 0.05 was considered statistically significant.

## Results

### Baseline characteristics of the training and validation cohorts

**Fig. 2:**
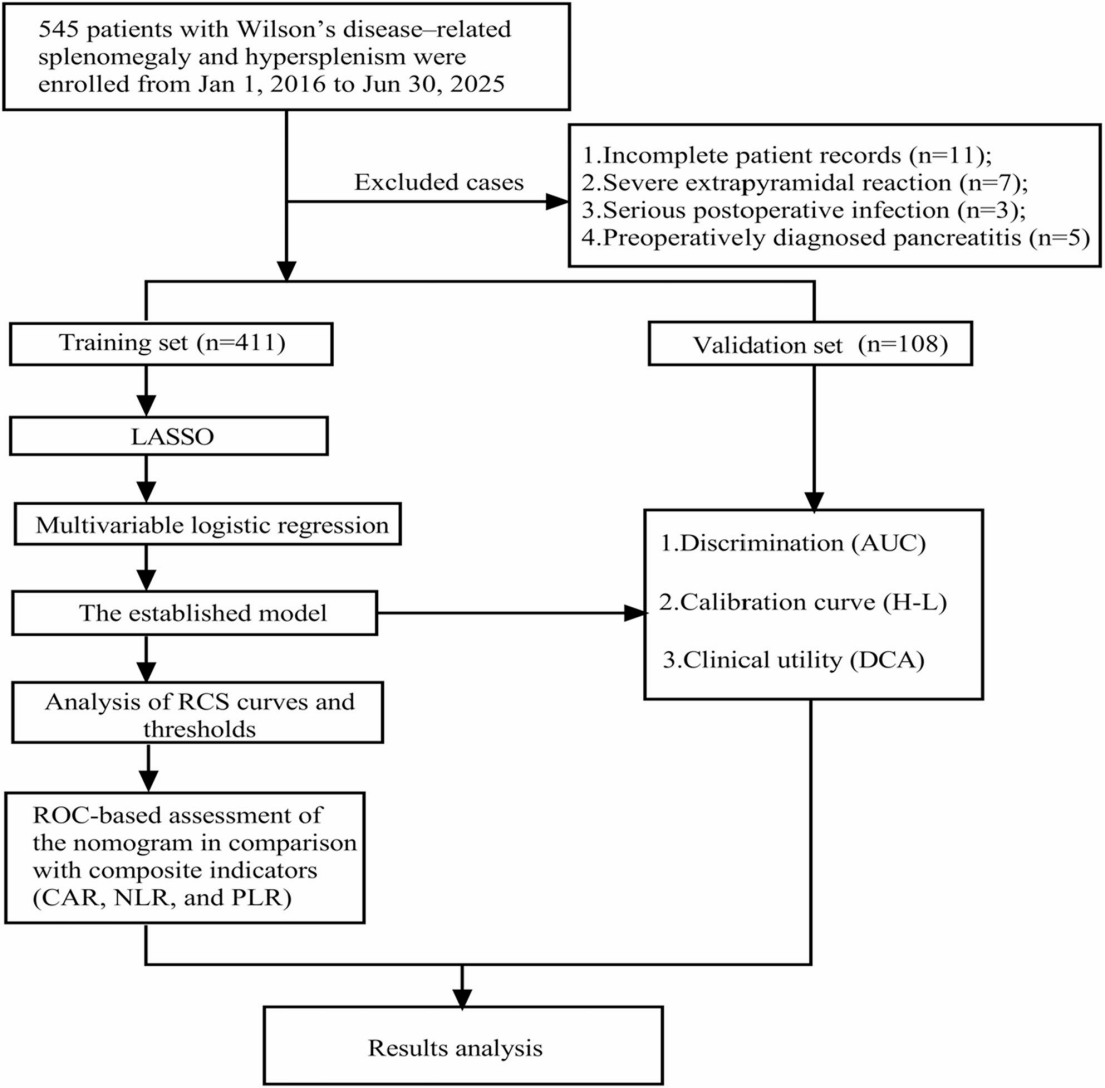
Flowchart of patient selection and study design. Notes: This figure illustrates the enrollment of 519 patients, divided into training (*n* = 411) and validation (*n* = 108) cohorts for model development. Abbreviations: LASSO: Least Absolute Shrinkage and Selection Operator; AUC: Area Under the Curve; H-L: Hosmer-Lemeshow (a goodness-of-fit test); DCA: Decision Curve Analysis; CAR: C-reactive protein to albumin ratio; NLR: Neutrophil-to-lymphocyte ratio; PLR: Platelet-to-lymphocyte ratio

To evaluate the applicability and robustness of the predictive model, Fig. 2 depicts the enrollment of 519 patients, who were allocated to a training cohort (*n* = 411) and a validation cohort (*n* = 108). As shown in Table 1, there were no statistically significant differences between the two cohorts in the comprehensive range of clinical records, Surgical details and surgical variables. These included age, gender, Child-Pugh grade, BMI, diabetes, hypertension, smoking status, history of abdominal surgery, splenomegaly grade, pancreatic attenuation, MPD diameter, PVD, SVD, PVV, hematologic indices, biochemical parameters, surgical approach, methods of splenic hilum management, splenic hilum division type, intraoperative blood loss, operative duration, incidence of POPF, and CDC (all *P* > 0.05). The overall balance in baseline characteristics confirms the comparability of the two cohorts for subsequent model development and validation.

**Table 1:** Comparison of baseline demographic and clinical characteristics between the training and validation cohorts

Characteristic	Overall (*n* = 519)	Training set (*n* = 411)	Validation set (*n* = 108)	t/z/χ2	*p* value
Clinical records					
Age (years)	27.00 (24.00–31.00)	27.00 (24.00–32.00)	27.00 (23.00–31.00)	1.418	0.234
Gender, n (%)				1.661	0.197
Female	240 (46.24)	196 (47.69)	44 (40.74)		
Male	279 (53.76)	215 (52.31)	64 (59.26)		
Child-Pugh, n (%)				2.670	0.102
A	418 (80.54)	337 (82.00)	81 (75.00)		
B	101 (19.46)	74 (18.00)	27 (25.00)		
BMI (kg/m^2^)	24.00 (21.00–28.00)	24.00 (21.00–28.00)	25.00 (21.00–29.00)	1.249	0.264
Diabetes, n (%)				0.369	0.544
No	427 (82.27)	336 (81.75)	91 (84.26)		
Yes	92 (17.73)	75 (18.25)	17 (15.74)		
Hypertension, n (%)				1.178	0.278
No	462 (89.02)	369 (89.78)	93 (86.11)		
Yes	57 (10.98)	42 (10.22)	15 (13.89)		
Smoking history, n (%)				0.027	0.869
No	359 (69.17)	285 (69.34)	74 (68.52)		
Yes	160 (30.83)	126 (30.66)	34 (31.48)		
Abdominal surgery, n (%)				0.140	0.708
No	287 (55.30)	229 (55.72)	58 (53.70)		
Yes	232 (44.70)	182 (44.28)	50 (46.30)		
Splenomegaly grade, n (%)				1.588	0.452
Ⅰ	195 (37.57)	149 (36.25)	46 (42.59)		
Ⅱ	171 (32.95)	137 (33.33)	34 (31.48)		
Ⅲ	153 (29.48)	125 (30.41)	28 (25.93)		
Pancreatic_attenuation, n (%)				0.127	0.722
< 30 HU	242 (46.63)	190 (46.23)	52 (48.15)		
≥ 30 HU	277 (53.37)	221 (53.77)	56 (51.85)		
MPD_diameter, n (%)				2.251	0.134
≤ 3 mm	217 (41.81)	165 (40.15)	52 (48.15)		
> 3 mm	302 (58.19)	246 (59.85)	56 (51.85)		
PVD (mm)	14.92 ± 3.11	15.02 ± 3.17	14.55 ± 2.85	1.998	0.158
SVD (mm)	12.09 ± 2.77	12.09 ± 2.88	12.13 ± 2.30	0.025	0.875
PVV (cm/s)	15.20 (13.35–16.90)	15.20 (13.30–17.00)	15.20 (13.75–16.50)	0.038	0.845
WBC (×10^9^/L)	3.50 (3.06–3.90)	3.51 (3.02–3.99)	3.40 (3.10–3.70)	1.582	0.209
RBC (×10^12^/L)	3.04 ± 0.47	3.05 ± 0.49	3.01 ± 0.40	0.708	0.401
PLT (×10^9^/L)	95.00 (83.00–106.00.00.00)	96.00 (81.00–110.00.00.00)	93.50 (89.00–99.00)	0.632	0.427
D-dimer (mg/L)	0.61 (0.50–0.72)	0.61 (0.46–0.74)	0.61 (0.55–0.66)	0.337	0.562
AST (U/L)	40.52 ± 7.56	40.30 ± 7.77	41.34 ± 6.67	1.629	0.202
ALT (U/L)	42.00 (36.00–47.00)	42.00 (35.00–47.00)	42.00 (38.00–46.00)	0.085	0.770
ALB (g/L)	36.00 (32.00–40.50.00.50)	36.00 (31.00–41.00)	35.00 (33.00–39.00)	0.638	0.424
TBIL (µmmol/L)	26.35 ± 6.17	26.41 ± 6.65	26.09 ± 3.85	0.229	0.632
BUN (mmol/L)	6.08 ± 1.53	6.05 ± 1.63	6.19 ± 1.07	0.676	0.411
Cr (µmol/L)	81.22 (73.09–87.84)	80.29 (70.58–89.02)	82.72 (79.69–85.52)	3.672	0.055
Blood amylase (U/L)	52.11 (47.98–56.61)	52.38 (46.83–57.44)	51.64 (49.51–53.56)	0.303	0.582
CP (mg/L)	96.00 ± 8.75	96.14 ± 9.42	95.48 ± 5.53	0.482	0.488
U-Cu (µg/d)	213.86 ± 14.21	213.75 ± 13.74	214.30 ± 15.93	0.127	0.722
S-Cu (µg/dL)	45.28 ± 4.82	45.22 ± 5.08	45.48 ± 3.71	0.237	0.627
Surgical details					
Operation method, n (%)				1.210	0.271
LS	83 (15.99)	62 (15.09)	21 (19.44)		
OS	436 (84.01)	349 (84.91)	87 (80.56)		
Hilum_management, n (%)				2.384	0.123
ED	236 (45.47)	194 (47.20)	42 (38.89)		
NED	283 (54.53)	217 (52.80)	66 (61.11)		
Hilum_division, n (%)				0.102	0.749
Ⅰ	238 (45.86)	187 (45.50)	51 (47.22)		
Ⅱ	281 (54.14)	224 (54.50)	57 (52.78)		
Postoperative Information					
Operation time (min)	203.00 (197.00–209.00.00.00)	202.00 (196.00–208.50.00.50)	205.00 (201.00–211.00.00.00)	8.231	0.051
Intraoperative_hemorrhage (mL)	221.00 (205.00–237.00.00.00)	222.00 (199.00–241.00.00.00)	220.00 (213.00–225.00.00.00)	1.139	0.286
POPF, n (%)				0.119	0.731
No	424 (81.70)	337 (82.00)	87 (80.56)		
Yes	95 (18.30)	74 (18.00)	21 (19.44)		
CDC, n (%)				0.700	0.978
Ⅰ	432 (83.24)	343 (83.45)	89 (82.41)		
Ⅱ	52 (10.02)	40 (9.73)	12 (11.11)		
Ⅲ	24 (4.62)	19 (4.62)	5 (4.63)		
Ⅳ	7 (1.35)	6 (1.46)	1 (0.93)		
Ⅴ	4 (0.77)	3 (0.73)	1 (0.93)		

The data are expressed as mean ± standard deviation, median (Q1–Q3), or number (percentage)

Abbreviations: *BMI* Body mass index, *PVD* Portal vein diameter, *SVD* Splenic vein diameter, *PVV* Portal vein velocity, *WBC* White blood cell, *RBC* Red blood cell, *PLT* Platelet, *AST* Aspartate aminotransferase, *ALB* Albumin, *ALT* Alanine aminotransferase, *TBIL* Total bilirubin, *BUN* Blood urea nitrogen, *Cr* Creatinine, *CP* Ceruloplasmin, *S-Cu* Serum copper, *U-Cu* Urine copper, *CDC* Clavien–Dindo classification, *MPD* Main pancreatic duct, *POPF* Postoperative pancreatic fistula, *LS* Laparoscopic splenectomy, *OS* Open splenectomy, *ED* Energy device, *NED* Non-energy device

### Variables selection by LASSO regression

**Fig. 3:**
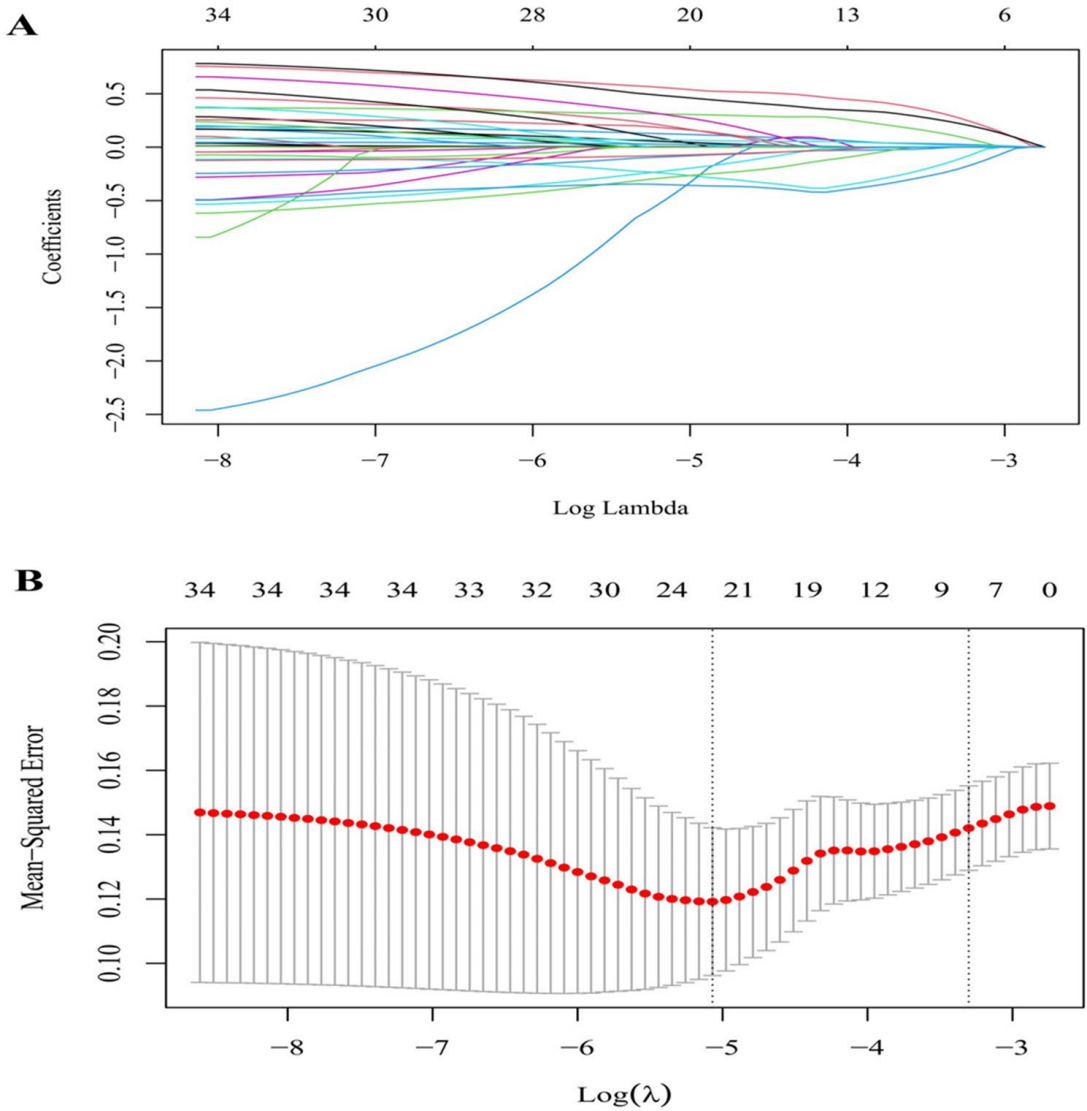
LASSO regression and 10-fold cross-validation. Notes: **A** Coefficient profiles for candidate variables across varying log (λ) values, demonstrating progressive shrinkage of coefficients toward zero as λ increases. **B** Mean squared error (MSE) curve from 10-fold cross-validation; the left dashed vertical line represents the λ yielding the minimum MSE, and the right dashed vertical line denotes the λ selected according to the one-standard-error (1-SE)

Optimization of predictors for POPF was performed using LASSO regression with 10-fold cross-validation. The tuning parameter λ was determined according to the minimum deviance plus one standard error (1-SE) criterion, yielding an optimal λ of 0.037. This analysis identified seven variables with non-zero coefficients: CDC, MPD diameter, BMI, history of abdominal surgery, type of splenic hilum division, splenomegaly grade, and operation time (Fig. 3). The resulting parsimonious model minimized overfitting while maintaining diagnostic performance through selective feature retention.

### Multivariate logistic regression analysis and model construction

To minimize potential confounding and account for collinearity among variables, a multivariable logistic regression analysis was performed. The analysis identified the following independent risk factors for POPF, including operation time, history of abdominal surgery, BMI, splenomegaly grade (III), and CDC grade. Conversely, splenic hilum division (type II) and MPD diameter > 3 mm were protective factors (Table 2). The final predictive model was expressed as: *Logit (P)* = − 12.041 + 0.038 × Operation time (min) + 0.799× (Abdominal surgery: Yes) – 0.705 × (Splenic hilum division: II) + 0.075 × BMI (kg/m^2^) + 0.368× (Splenomegaly grade: Ⅱ) + 0.925 × (Splenomegaly grade: III) – 0.675 × (MPD diameter > 3 mm) + 0.546 × CDC (grade). The model achieved a minimum Akaike Information Criterion (AIC) of 345.49, indicating optimal goodness-of-fit. This equation was subsequently applied to calculate the probability of POPF occurrence.

**Table 2:** Multivariate logistic regression of predictor variables

Types	β	SE	OR (95%CI)	Wald	*P*
Operation time (min)	0.038	0.011	1.039 (1.017–1.062)	3.471	0.001
Abdominal surgery, n (%)					
No	*Reference*				
Yes	0.799	0.287	2.223 (1.266–3.901)	2.781	0.005
Hilum division, n (%)					
Ⅰ	*Reference*				
Ⅱ	−0.705	0.285	0.494 (0.283–0.864)	−2.447	0.013
BMI (kg/m^2^)	0.075	0.029	1.078 (1.018–1.141)	2.563	0.010
Splenomegaly grade, n (%)					
Ⅰ	*Reference*				
Ⅱ	0.368	0.360	1.445 (0.713–2.925)	1.023	0.306
Ⅲ	0.925	0.348	2.521 (1.275–4.987)	2.659	0.008
MPD diameter, n (%)					
≤ 3 mm	*Reference*				
> 3 mm	−0.675	0.282	0.509 (0.293–0.885)	−2.392	0.017
CDC (grade)	0.546	0.174	1.726 (1.228–2.428)	3.145	0.002

Abbreviations: β regression coefficient, *SE* Standard error, *OR* Odds ratio, *CI* Confidence interval, *CDC* Clavien–dindo classification, *MPD* Main pancreatic duct

### Predictive nomogram for POPF using clinical factors and SHAP analysis after splenectomy for WD

**Fig. 4:**
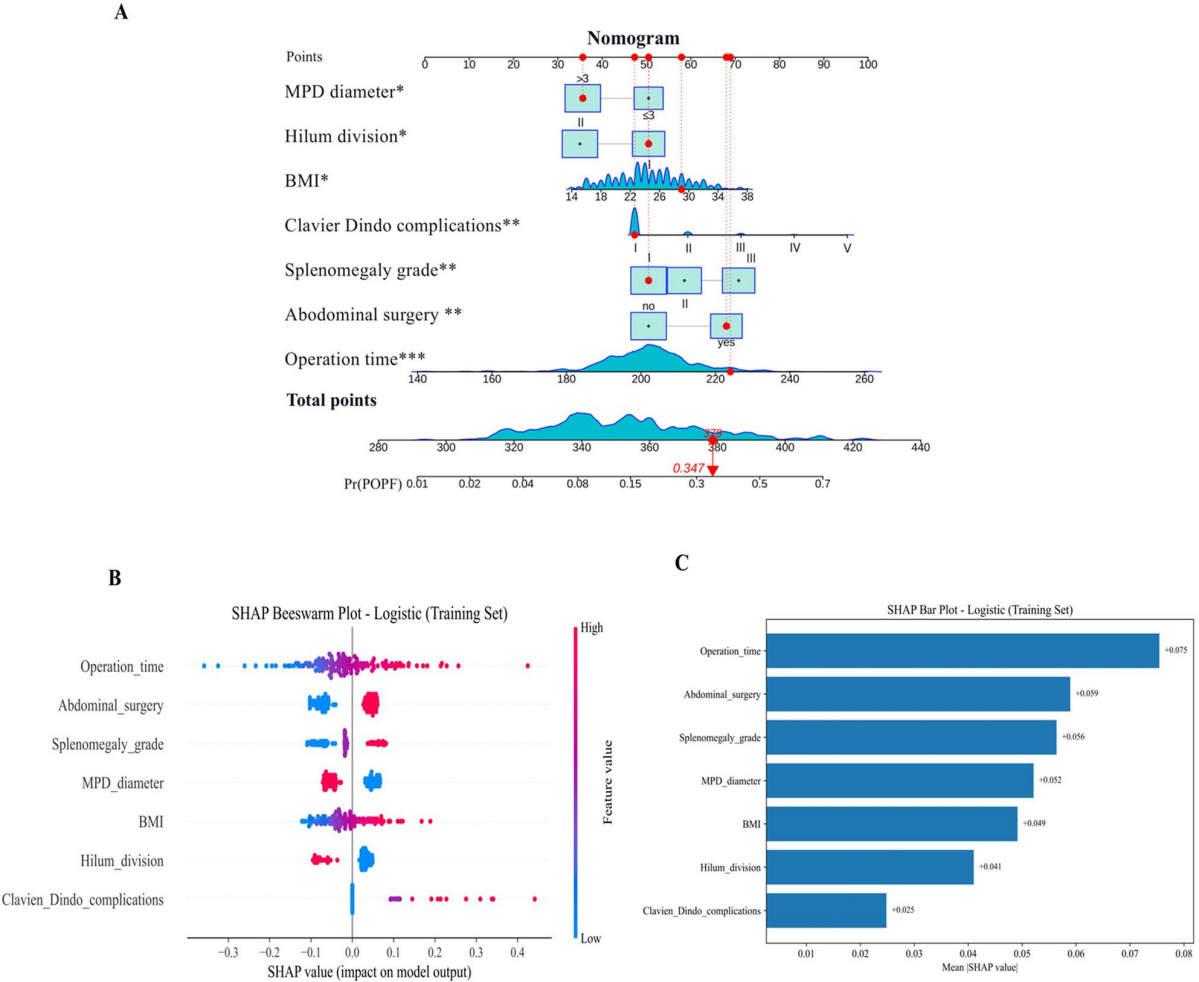
Development of POPF predictive model using nomogram and SHAP analysis. Notes: 1. The nomogram, derived from multivariable logistic regression, enables individualized risk estimation by summing the points assigned to each predictor variable. The cumulative point score corresponds to the predicted probability of postoperative pancreatic fistula (POPF). As an example, Patient ID 3, who underwent splenectomy, had a calculated risk of 34.7% for developing POPF

**Fig. 5:**
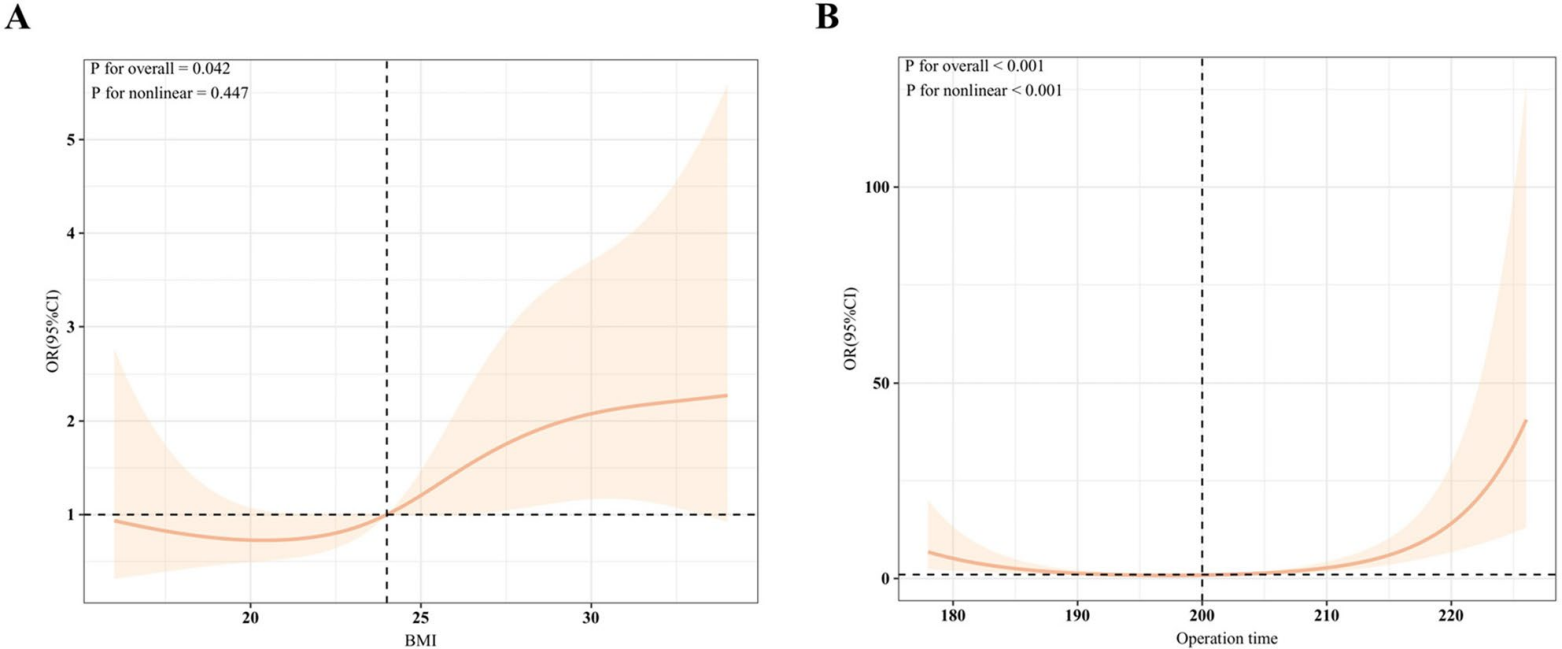
RCS curves for the effects of BMI and operation time on POPF. Notes: OR were ajusted for abdominal surgery, hilum division, splenomegaly grade, MPD diameter, CDC, and operation time or BMI. The solid line shows the estimates, with the orange-yellow area indicating 95% confidence intervals (CI). Abbreviations: OR (95%CI): Odds Ratio (95% Confidence Interval); BMI: Body Mass Index

To demonstrate the predictive efficacy of a multivariable logistic regression model in evaluating multiple clinical variables on the probability of POPF occurrence, Fig. 4A presented a nomogram that integrated variables including MPD diameter, hilum division, BMI, Clavien-Dindo complications, splenomegaly grade, abdominal surgery, and operation time to generate a predictive model for POPF. Based on the nomogram, an individualized scoring system enabled the calculation of the POPF probability for each patient. Fig. 4B, a SHAP beeswarm plot, illustrated the extent and directionality of the impact of various clinical features on the model’s predictive outcomes. The results indicated that operation time and abdominal surgery exerted the greatest influence on POPF prediction, both showing notable positive effects (SHAP value > 0). In contrast, MPD diameter and hilum division contributed negatively to the model output (SHAP value < 0). Fig. 5C, a SHAP bar plot, further quantified the average contribution of each variable to the POPF occurrence probability. It was evident that operation time (+ 0.075) had the most significant impact on POPF probability, followed by abdominal surgery (+ 0.059) and splenomegaly grade (+ 0.056). Although MPD diameter (+ 0.052), BMI (+ 0.049), hilum division (+ 0.041), and Clavien-Dindo complications (+ 0.025) also influenced the model, their effects were comparatively minor. These findings suggested that operation time and abdominal surgery were the primary factors in predicting POPF, and the combination of the nomogram and SHAP analysis effectively assessed the risk of POPF occurrence.

In Fig. 5B, the colour of the SHAP values denotes the magnitude of the feature values, with blue representing low values and red indicating higher ones.

### Nonlinear association analysis of BMI and operation time on the risk of POPF

This study employed RCS analysis to reveal the nonlinear associations between BMI, operation time, and the risk of POPF (Fig. 5).The RCS curve is helpful in identifying different inflection points, clarifying the trend of POPF risk beyond these thresholds, thereby demonstrating a threshold effect and enhancing model accuracy. Threshold effect analysis (Table 3) demonstrated that, in the conventional linear regression model, BMI and operation time were positively correlated with elevated POPF risk (OR: 1.078, 95% CI: 1.018–1.141, *P* = 0.010 and OR: 1.039, 95%CI: 1.017–1.062, *P* = 0.001). In the two-piecewise linear regression model, the inflection point for BMI was 24 kg/m^2^ (OR: 1.043 [< 24], *P* = 0.538; OR: 1.100 [> 24], *P* = 0.046). For operation time, the inflection point was 200 min (OR: 0.897 [< 200], *P* < 0.001; OR: 1.175 [> 200], *P* < 0.001), with a significant likelihood ratio test (*P* < 0.001). These findings underscore that BMI exceeding 24 kg/m^2^ and operation times surpassing 200 min represent pivotal thresholds for augmented POPF risk, thereby informing preoperative risk stratification and surgical optimization to mitigate complications of POPF after splenectomy.

**Table 3:** Threshold effect analysis of factors related to the occurrence of POPF

POPF	OR (95%CI)	*P* value
BMI (kg/m^2^)		
Model 1 Fitting model by standard linear regression	1.078 (1.018–1.141)	0.010
Model 2 Fitting model by two-piecewise linear regression		
Inflection point	24	
< 24	1.043 (0.917–1.199)	0.538
> 24	1.100 (1.002–1.208)	0.046
P for likelihood ratio test		0.592
Operation time (min)		
Model 1 Fitting model by standard linear regression	1.039 (1.017–1.062)	0.001
Model 2 Fitting model by two-piecewise linear regression		
Inflection point	200	
< 200	0.897 (0.852–0.94)	< 0.001
> 200	1.175 (1.126–1.23)	< 0.001
P for likelihood ratio test		< 0.001

This analysis was adjusted for various factors, including abdominal surgery, hilum division, splenomegaly grade, MPD diameter, CDC, and operation time or BMI

Abbreviations: *POPF* Postoperative pancreatic fistula, *OR (95%CI)* Odds Ratio (95% Confidence Interval), *BMI* Body Mass Index

### Discrimination performance of the predictive model

**Fig. 6:**
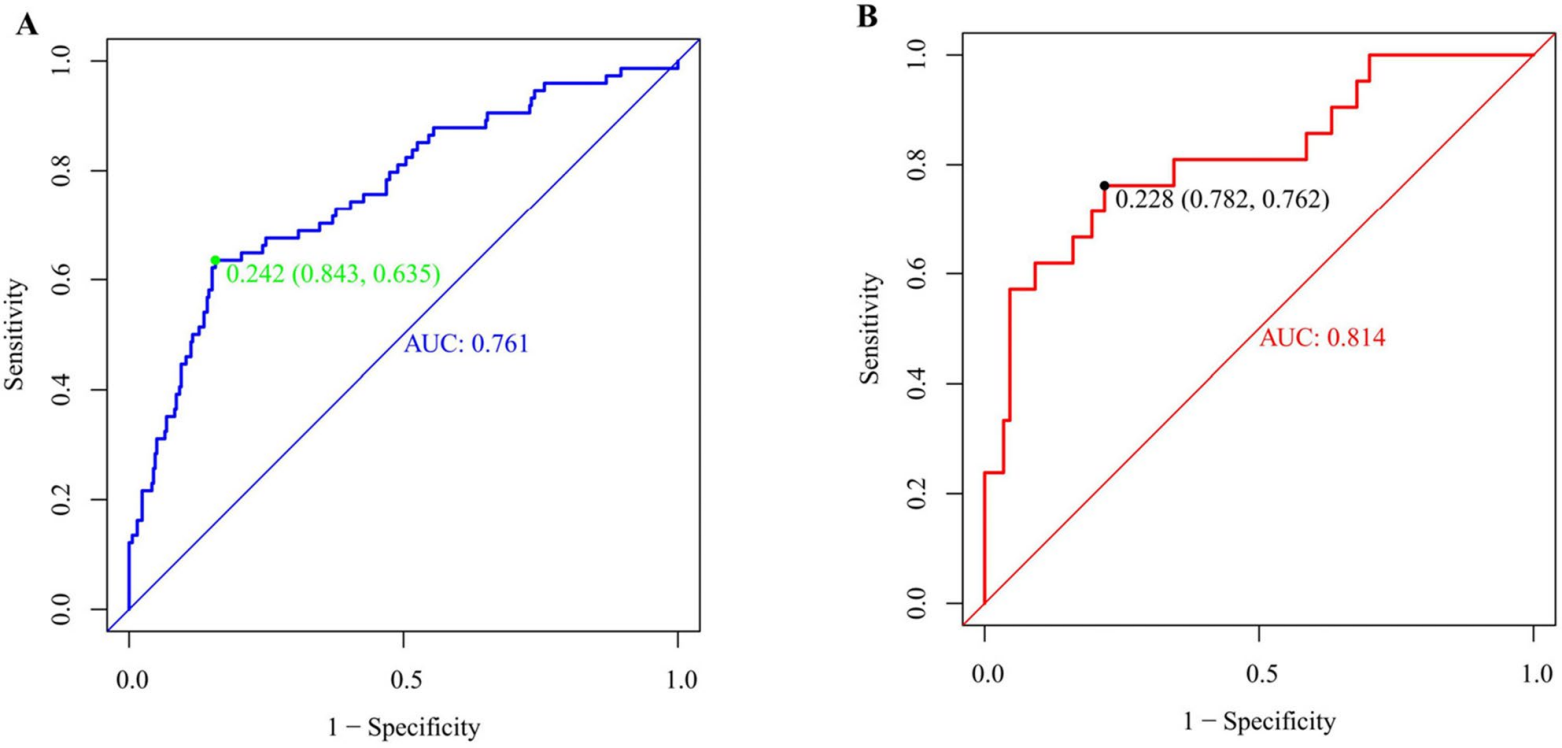
The ROC curve of the nomogram for predicting POPF after splenectomy for WD. Notes: For both panels, the predicted probability was calculated using the logistic transformation: Probability = exp (Logit P)/[1 + exp (Logit P)], derived from the training cohort model. Discriminative performance was evaluated with receiver operating characteristic (ROC) curve analysis, applied to the training cohort (Figure **A**) and validation cohort (Figure **B**), with predicted probability serving as the key performance metric

The discriminative performance of the predictive model was assessed using ROC curve analysis. In the training cohort (Fig. 6A), the model yielded an area under the curve (AUC) of 0.761 (95% CI: 0.697–0.825). At the optimal cutoff value of 0.242, sensitivity and specificity were 63.51% and 80.54%, respectively. In the validation cohort (Fig. 6B), the AUC was 0.814 (95% CI: 0.702–0.927), with an optimal cutoff of 0.228 providing a sensitivity of 78.16% and a specificity of 76.19%. Collectively, these findings demonstrate that the nomogram maintained strong discriminative ability across both datasets, with particularly high predictive accuracy in the validation cohort.

### Calibration of the predictive model

**Fig. 7:**
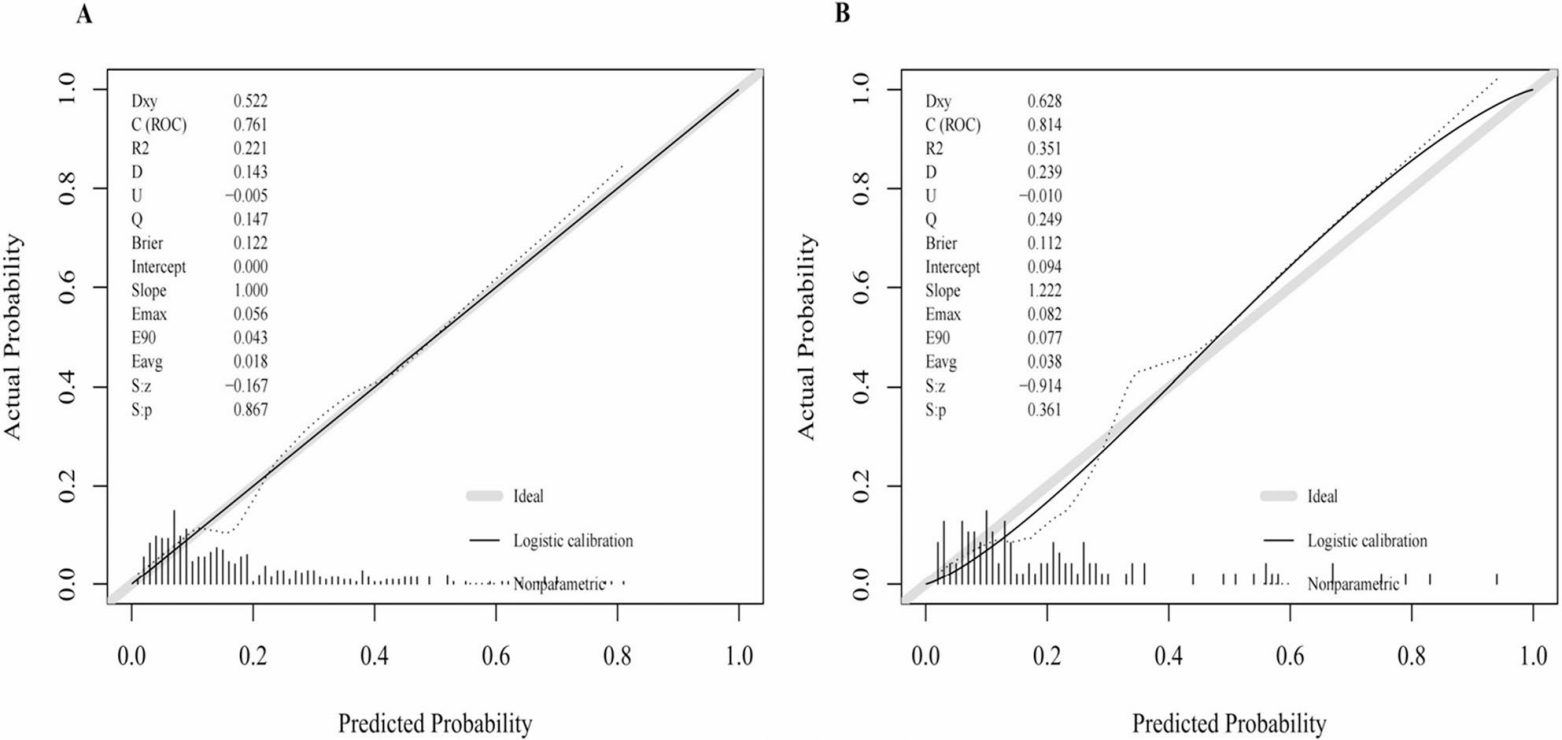
Calibration curves of the nomogram in the training (**A**) and validation (**B**) cohorts. Notes: The x-axis shows predicted probabilities and the y-axis shows observed probabilities. The gray diagonal line represents the ideal reference line, the solid line indicates the logistic regression–based calibration curve, and the dashed line denotes the nonparametric smooth fit

Model calibration was assessed using calibration plots and the HL goodness-of-fit test. The nomogram showed close agreement between predicted probabilities and observed incidence in both the training and validation cohorts, as shown in Fig. 7. The HL test yielded χ² = 6.289 (*P* = 0.615) for the training cohort and χ² = 8.240 (*P* = 0.410) for the validation cohort, indicating no significant lack of fit and satisfactory calibration. These findings demonstrate that the model possesses robust stability and strong generalizability across both cohorts, supporting its reliability for predicting the risk of POPF.

### Clinical utility of the predictive model

**Fig. 8:**
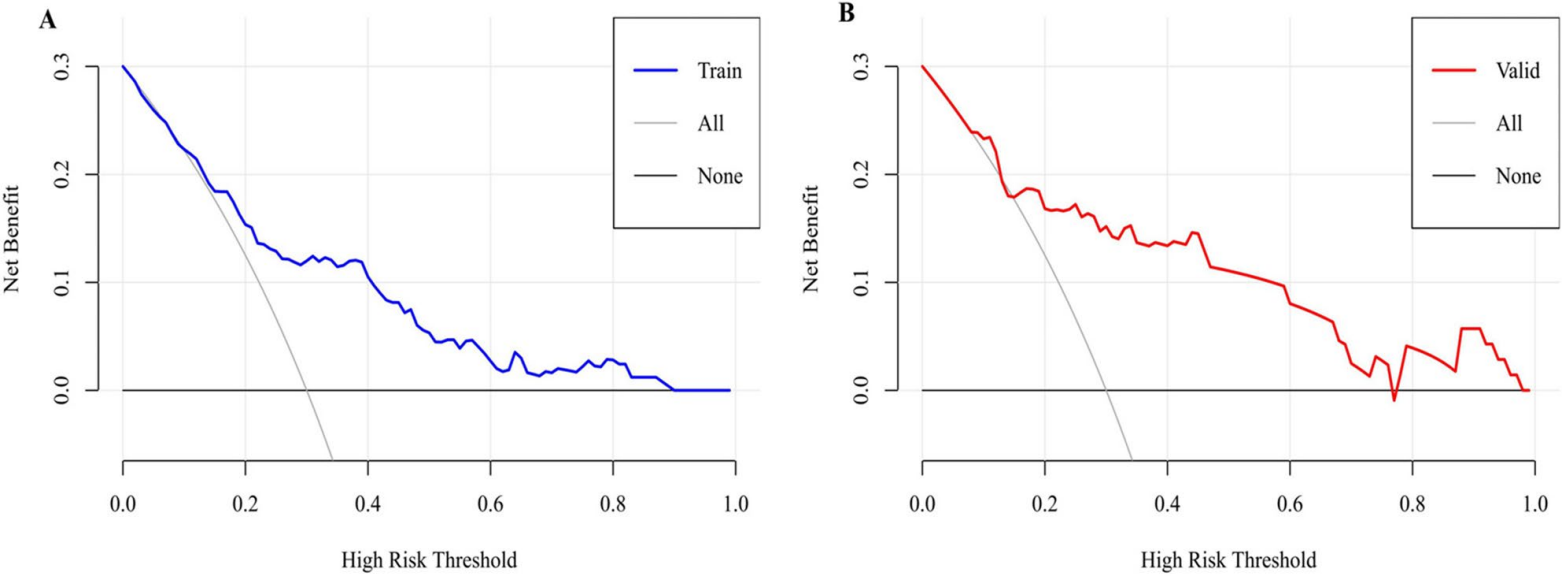
DCA of the nomogram in the training (**A**) and validation (**B**) cohort. Notes: The x-axis indicates the threshold probability for defining high-risk patients, and the y-axis represents the net benefit. The model (blue/red line) demonstrates superior net benefit compared with the “treat-all” (gray line) and “treat-none” (black line) strategies across most threshold ranges

To further explore the clinical applicability of the nomogram, DCA was conducted. As illustrated in Fig. 8, the model provided a meaningful net benefit across a wide range of threshold probabilities, specifically 12%–91% in the training cohort and 15%–98% in the validation cohort. These findings highlight the nomogram’s robust clinical utility, supporting its potential role as a decision-support tool to guide individualized intervention strategies.

### Comparative performance of the nomogram and composite indices

**Fig. 9:**
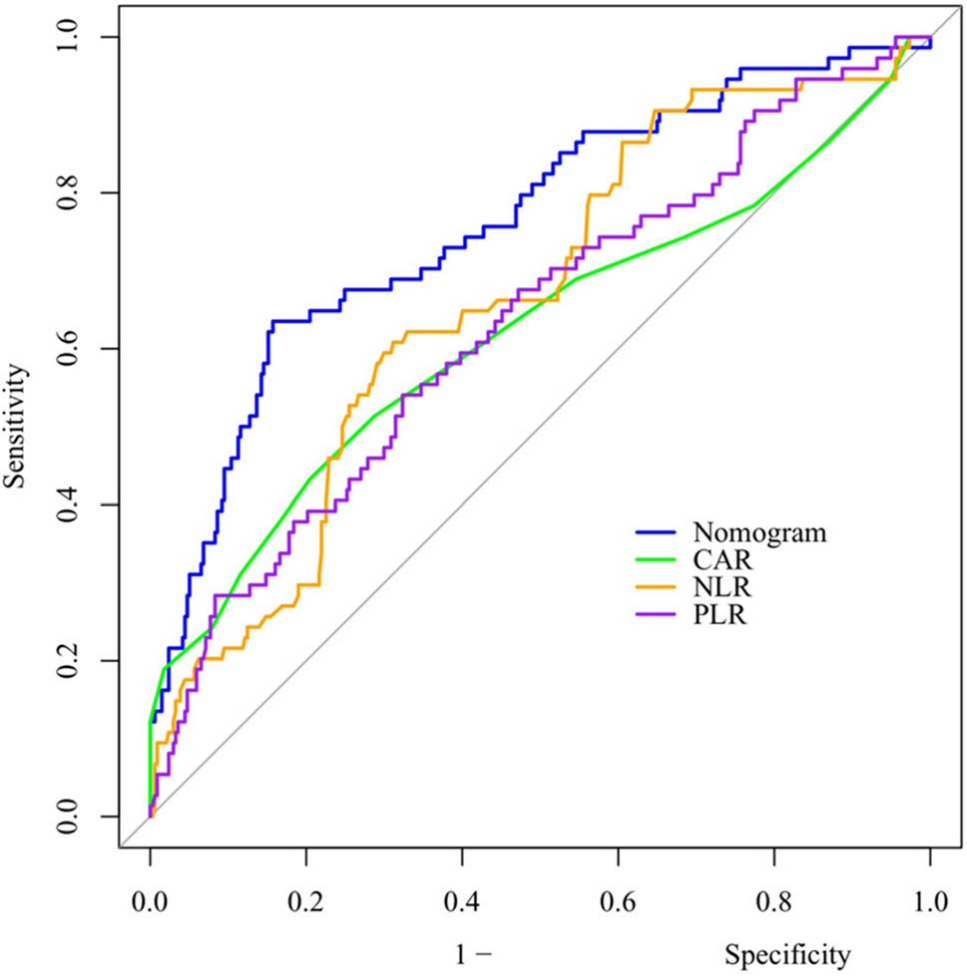
ROC curves of the nomogram versus preoperative composite indicators for predicting POPF after splenectomy. Notes: ROC curve comparison indicates that the blue line (nomogram) exhibits the largest area under the curve, consistently outperforming the composite indicators CAR (green), NLR (orange), and PLR (violet) in discriminatory accuracy

The predictive performance of the nomogram in comparison with preoperative composite indicators (CAR, NLR, and PLR) for assessing the risk of post-splenectomy pancreatic fistula in WD was evaluated using ROC curve analysis. As shown in Table 4, the nomogram yielded an AUC of 0.761 (95% CI: 0.697–0.825) at an optimal cutoff value of 0.242, demonstrating significantly superior discriminative power relative to CAR (AUC = 0.621; Z = 2.786, *P* = 0.005), NLR (AUC = 0.662; Z = 2.067, *P* = 0.038), and PLR (AUC = 0.633; Z = 2.590, *P* = 0.009). These findings underscore the enhanced predictive accuracy of the nomogram for this clinical endpoint (Fig. 9).

**Table 4:** ROC curve analysis comparing the nomogram model and preoperative composite indices in POPF after splenectomy for WD

Projects	AUC	95%CI	Sensitivity (%)	Specificity (%)	Cut-off value	PPV	NPV
Nomogram	0.761	0.697–0.825	0.635	0.842	0.242	0.470	0.913
CAR	0.621	0.542–0.702	0.432	0.795	0.105	0.317	0.865
NLR	0.662	0.595–0.729	0.608	0.688	3.585	0.300	0.889
PLR	0.633	0.561–0.705	0.541	0.677	158.785	0.268	0.870

Abbreviations: *CAR* C-reactive protein to Albumin Ratio, *NLR* Neutrophil to Lymphocyte Ratio, *PLR* Platelet to Lymphocyte Ratio, *PPV* Positive Predictive Value, *NPV* Negative Predictive Value

## Discussion

Splenectomy continues to represent a crucial therapeutic strategy for patients with WD complicated by portal hypertension. Clinically, the operation not only alleviates splenic artery steal syndrome and enhances hepatic function [19, 20], but also corrects hypersplenism-associated pancytopenia-including anemia, leukopenia, and thrombocytopenia-thereby enabling long-term anti-copper therapy [21]. Nevertheless, due to the intricate regional anatomy, where the pancreatic tail lies adjacent to the stomach, duodenum, bile duct, spleen, and celiac axis, this structure is particularly susceptible to injury during splenic hilar dissection, predisposing patients to POPF. Once present, POPF can lead to intra-abdominal hemorrhage, infection, abscess formation, and gastrointestinal fistulae, and may become life-threatening in severe scenarios [22, 23]. While most existing research has concentrated on POPF following pancreatoduodenectomy, studies addressing its occurrence after splenectomy remain limited. Therefore, identifying predictive factors for POPF in this context and constructing a reliable risk model hold significant clinical importance.

According to the 2016 ISGPS definition, when both biochemical leak (BL) and clinically relevant POPF (Grades B/C) are considered, studies involving splenectomy have reported an overall incidence ranging from 14% to 24% [24]. Mehdorn et al. observed a total rate of 14.6% (10.1% BL, 4.5% Grades B/C). More recent single-center series restricted to isolated splenectomy documented rates up to 23.7%, with 5% classified as Grade B events [22, 24]. In the present retrospective study involving patients with WD complicated by splenomegaly and hypersplenism, the overall incidence of POPF (including BL) was 18.3%. Specifically, BL occurred in 9.06% of cases, while grade B and grade C fistulas were observed in 7.51% and 1.73% of cases, respectively. This overall incidence falls between the rates reported in the two aforementioned categories of studies. These discrepancies may arise from differences in case composition (e.g., the proportion of primary versus secondary splenectomies), the routine use of postoperative drains, perioperative management strategies, and the diagnostic criteria applied.

Although the overall incidence of POPF has gradually declined, it continues to represent a critical determinant of postoperative recovery and long-term prognosis. A single-center retrospective study indicated that secondary splenectomy was an independent predictor of POPF, primarily attributable to distorted splenic hilar anatomy and dense adhesions that complicate dissection [8, 25]. In line with these findings, our analysis also identified prior abdominal surgery as a significant risk factor, most likely because adhesions obscure natural anatomical planes, necessitate extensive traction and reliance on energy-based hemostatic devices, and thereby heighten the likelihood of pancreatic-tail injury with delayed leakage as a consequence.

Furthermore, a 2025 systematic review revealed that, in models designed for POPF risk stratification after pancreatoduodenectomy, MPD diameter was the most commonly incorporated variable, included in nearly 80% of models, followed by pancreatic texture (51.4%) and body mass index (BMI, 45.7%) [26]. Our observations align with these mechanistic insights: an increase in BMI is positively associated with the risk of POPF, as demonstrated by the RCS curve for BMI, which reveals a threshold value of 24.0 kg/m². Conversely, an MPD diameter exceeding 3 mm appears to confer a protective effect. Two plausible explanations can be proposed: (i) excessive visceral fat limits surgical exposure, necessitating repeated traction and prolonged use of energy devices, which in turn heightens tissue injury; (ii) although isolated splenectomy does not directly manipulate the pancreatic duct, the pancreatic tail or regions adjacent to the duct may sustain damage from traction and manipulation due to their anatomical proximity to the splenic hilum. A larger MPD suggests reduced pancreatic juice pressure within the ductal lumen, leading to lower resistance to intestinal drainage. In contrast, patients with a narrower MPD are more susceptible to injury of the pancreatic body/tail and accessory ducts following traction, thereby promoting persistent pancreatic fluid extravasation through the damaged sites [27, 28]. 

Several investigations have proposed that increased splenic volume or weight correlates with higher morbidity, including POPF, whereas other systematic reviews and single-center analyses failed to validate splenic size as an independent predictor [29, 30]. We endorse the former viewpoint, as our study demonstrates that the grade of splenomegaly is a significant risk factor for POPF. Severe splenomegaly typically results in short and thick splenic hilar vessels, coupled with limited surgical visibility. Under these conditions, dissection of the short gastric vessels and splenic vein branches often occurs in close proximity to the pancreatic tail, thereby increasing the risk of pancreatic injury. In contrast, the Type II splenic hilum division technique employed in our study-which emphasizes stepwise vascular control-combined with careful exposure of the “pancreatic tail-splenic hilum” region, is associated with a lower incidence of POPF. This association may highlight the importance of individual vessel ligation and precise anatomical dissection. 

This study further revealed that prolonged operation time was significantly associated with Clavien-Dindo grade ≥ II complications and the occurrence of postoperative pancreatic fistula (POPF), particularly when the operation time exceeded 200 min, underscoring the need for heightened vigilance. Mehdorn et al. reported that prolonged operative time served as an independent predictor of POPF following splenectomy, highlighting the influence of intraoperative complexity and tissue trauma [8]. Similarly, Kwiatkowska et al. demonstrated that intraoperative hemorrhage-considered a surrogate for technical difficulty-was linked to higher Clavien-Dindo classifications [31]. Taken together, prolonged operative duration and severe complications reflect heightened systemic inflammation and local tissue injury, which may facilitate the progression from biochemical leakage to clinically relevant POPF, in accordance with our multivariable findings. 

Against the backdrop of these risk determinants, recent efforts have focused on easily measurable inflammatory composite indices for improved prediction. In pancreatoduodenectomy, Funamizu N et al. demonstrated that preoperative CAR achieved an AUC of 0.888 (cutoff 0.09) for forecasting POPF [32], while other investigations reported more modest AUCs of 0.71 and 0.72 for NLR and PLR, respectively [33]. Within our surgical context of splenectomy for WD, however, the nomogram clearly outperformed these single indices, attaining an AUC of 0.761 (95% CI: 0.697–0.825) in the training cohort and significantly exceeding CAR, NLR, and PLR. These results highlight heterogeneity in POPF pathogenesis and risk profiles across operations, underscoring the necessity of population- and procedure-specific predictive models. 

Based on the factors contributing to pancreatic fistula, we have drawn two main lessons. On one hand, a comprehensive, full-spectrum treatment protocol should be established for surgical patients, which includes: preoperative comprehensive assessment and optimization of surgical history, BMI (≥ 24 kg/m²), splenic size, pancreatic duct diameter via imaging, and other relevant parameters; intraoperative precise dissection of the splenic hilum, careful traction of the pancreatic tail, adoption of Type II splenic hilum division during splenectomy, and minimization of thermal injury to the pancreatic surface; and postoperative standardized differentiation and fixation of drains at various sites. Additionally, integrating risk thresholds (such as a predicted POPF risk > 20%) into the electronic medical record (EMR) alert system, and using this model for early identification of high-risk patient cohorts, is recommended. On the other hand, for this high-risk subgroup, preoperative prophylactic administration of protease inhibitors and somatostatin analogs is advised. Intraoperatively, the involvement of senior surgeons should be prioritized whenever possible, with operation time controlled to within 200 min and selective pancreatic stenting based on assessment. Postoperatively, extending the drainage duration, intensifying biochemical monitoring, and implementing targeted interventions can effectively reduce the incidence of pancreatic fistula. 

Nevertheless, several limitations merit consideration. First, owing to the retrospective design and reliance on multivariable modeling, selection bias cannot be excluded. Although external validation was conducted, all cases originated from centers within the same geographic region, thereby restricting generalizability. Second, dynamic postoperative variables known to influence POPF-such as POD1/3 drain amylase or CRP/CAR-were not incorporated, which may have underestimated the incremental predictive value of early clinical trajectories. Future investigations should therefore adopt prospective, multicenter designs with larger cohorts, incorporate serial postoperative biomarkers together with imaging and Doppler assessments, and evaluate whether risk-stratified interventions can effectively reduce POPF incidence and improve outcomes following splenectomy in WD. 

In conclusion, POPF is relatively common after splenectomy for WD, with an incidence of 18.3% observed in our cohort. Independent risk factors included history of abdominal surgery, BMI ≥ 24 kg/m², massive splenomegaly, prolonged operation time, and severe postoperative complications, whereas splenic hilum division (Type II) and MPD diameter > 3 mm served as protective factors. To mitigate surgical risks, particular emphasis should be placed on abdominal surgical history and operation time among the aforementioned influencing factors. Compared with conventional composite indicators (CAR, NLR, PLR), our nomogram provided superior discrimination and may support perioperative risk stratification and precision management, with the potential to reduce POPF‑related harm, maintain continuity of anti‑copper therapy, and improve outcomes.

## Supplementary Information

Supplementary Material 1.

## Data Availability

Data are available from the corresponding authors with ethics committee approval.
