# Supplementary material for: Risk factors and prediction model development for pancreatic fistula following splenectomy in Wilson’s disease patients with portal hypertension
Source: BMC Gastroenterol. 2025 Nov 25;25:884. doi: 10.1186/s12876-025-04475-w (PMC12751975; doi:10.1186/s12876-025-04475-w)
Supplement: Supplementary file 1 — Supplementary Material 1. [file 12876_2025_4475_MOESM1_ESM.doc]

| **Projects** | **Number of cases（%）** |
| --- | --- |
| Grade A (biochemical leak) | 47 (9.06) |
| Grade B | 39 (7.51) |
| Grade C | 9 (1.73) |

**Supplement 1. Grading and Incidence of Pancreatic Fistula**
